# Why hospital physicians attend work while ill? The spiralling effect of positive and negative factors

**DOI:** 10.1186/s12913-016-1802-y

**Published:** 2016-10-05

**Authors:** Fay Giæver, Signe Lohmann-Lafrenz, Lise Tevik Løvseth

**Affiliations:** 1 Department of Psychology, Norwegian University of Science and Technology, Trondheim, Norway; 2 Department of Occupational Medicine, St Olav University Hospital, Trondheim, Norway; 3 Department of Research and Development, Division of Mental Health Care, St Olav University Hospital, P box 3008 Lade, 7441 Trondheim, Norway

**Keywords:** Sickness presence, Physicians, Health, Stress, University hospital, Positive and negative causes and consequences, Intervention, Qualitative study

## Abstract

**Background:**

Recurrent reports from national and international studies show a persistent high prevalence of sickness presence among hospital physicians. Despite the negative consequences reported, we do not know a lot about the reasons why physicians choose to work when ill, and whether there may be some positive correlates of this behaviour that in turn may lead to the design of appropriate interventions. The aim of this study is to explore the perception and experience with sickness presenteeism among hospital physicians, and to explore possible positive and negative foundations and consequences associated with sickness presence.

**Methods:**

Semi-structured interviews of 21 Norwegian university hospital physicians.

**Results:**

Positive and negative dimensions associated with 1) evaluation of illness, 2) organizational structure, 3) organizational culture, and 4) individual factors simultaneously contributed to presenteeism.

**Conclusions:**

The study underlines the inherent complexity of the causal chain of events affecting sickness presenteeism, something that also inhibits intervention. It appears that sufficient staffing, predictability in employment, adequate communication of formal policies and senior physicians adopting the position of a positive role model are particularly important.

**Electronic supplementary material:**

The online version of this article (doi:10.1186/s12913-016-1802-y) contains supplementary material, which is available to authorized users.

## Background

Sickness presence, or presenteeism, refers to the situation of attending work while ill [1, 2]. Illness refers to the onset of an acute, episodic or chronic “health event” that involves severe, and less severe, physical conditions such as heart disease and stomach flu as well as psychological conditions such as depression. Studies show that presenteeism is particularly prevalent among physicians. A study by the Norwegian Medical Association’s show that 80 % of physicians have worked during an illness for which they would have sick-listed patients [3]. International studies report the same trend [4]. This occurs despite the fact that presenteeism has a wide range of negative consequences for physicians, other staff members and the organization as it affects recruitment and retention [5], workplace productivity and efficiency, and collaboration between professions and parts of the health services [6]. In addition, ill health among physicians affects the quantity and quality of patient care [7, 8], for example through the spread of contagious diseases to patients, medical errors, complaints and physician-patient communication, which in turn affect treatment adherence and patient recovery.

According to John’s model of presenteeism [1] the severity of the health event will dictate how influential personal or contextual factors will be in the employee’s decision to be absent or present. Accordingly, the potential adverse consequences of this behavior have contributed to the main focus being on its negative antecedents. Presenteeism among physicians as well as in the general population is associated with structural constraints and work-related determinants [9–12] such as difficulties with staff replacement, conflicting work demands, work pace and pressure, workload and job insecurity. The high prevalence of temporary positions, in particular among residents, in university hospitals can also contribute to presenteeism as physicians want to prove organizational commitment to achieve permanent employment [13].

In addition, individual and cultural factors can play a pivotal role in presenteeism. Johns [1] emphasized that certain organizations may be more liable to hold presenteeism cultures, particularly in the caring sector where professionals tend to be motivated by their loyalty to and concern for clients and patients. Hence, they are more likely to go to greater lengths when it comes to fulfilling work obligations. In a study where a private and a public hospital were compared [14] it was found that the hospital characterized by a “sanctuary culture” with a strong sense of loyalty to co-workers showed a prevalent adoption of teamwork-motivated sickness presence. In contrast, in the hospital that demonstrated more of a “battleground culture”, the employees’ professional identity and institutional loyalty were important triggers for sickness presence. Although the antecedents of sickness presence vary between contexts, studies from other contexts have also revealed similar presenteeism cultures. Simpson (1998), for instance, revealed that higher-level managers, predominantly men, adopted sickness presence as a way to achieve competitive advantage in a culture that demanded long work hours. Sickness presence can also emerge from a need to uphold a certain professional identity [15], which in turn leads to an unwillingness for physicians to adopt the patient role [16] and consequently to call in sick.

The current study derives from findings of a large quantitative study among European university hospital physicians (the Health and Organisation among University Hospitals in Europe, or HOUPE, study) that confirmed a persistent high prevalence of sickness presence [4] and that physicians typically hide or neglect their own illness and report a wide range of negative correlations such as role conflict, self-treatment and lack of organisational care. The study raised concerns about the apparent inability to change this behavior, despite increased knowledge about its negative consequences. This indicates that there is a need for a new and broader perspective on the correlates of presenteeism among physicians. Consequently, the mainly negative focus on sickness presence in the literature may have overlooked possible positive drivers for attending work when ill among employees who experience work as deeply meaningful and something that brings joy, energy and engagement. Absence is not necessarily the only remedy when recovering from illness. Maybe it is not a question of reducing sickness presence per se, but of focusing on the overall health and well-being of physicians through identifying when sickness presence is called for, how it can be arranged and when it should be avoided. It has been pointed out that physicians overall find their jobs meaningful, interesting and satisfying [17], and furthermore that aspects of their work such as unpredictability, high work pace and everyday challenges that are regarded as negative from an outsider’s perspective can be the very features that clinicians appreciate about their work and that make their work attractive [9, 18]. We need to explore the issue of presenteeism among physicians in more depth to understand when working whilst ill is perceived as negative or positive to the physician, the health services provided and consequently the patient, to enable interventions that benefit both parties.

The aim of the current study is to explore the perception and experiences of presenteeism among hospital physicians. Here, we wanted to focus on organisational, cultural and individual factors in particular, and to provide more in-depth knowledge about the contents of these factors in the daily life of hospital physicians. In addition, we wanted to open up positive as well as negative perspectives on attending work while ill, and the interaction between these factors.

## Methods

### Study design

The study was initiated as part of the HOUPE study and participants were recruited from one of the participating public university hospitals in a large city (population = 180,000) with emergency assignments within a health authority with an population of approximately 715,000 people. The study was approved by the administration and the medical association at the hospital, and the regional ethical committee (ref. 2013/1355). A total of 21 physicians working at the hospital were interviewed through the adoption of a semi-structured interview guide. All participants signed a written consent to participate prior to the interviews. None of the participants received any compensation to participate in the study.

### Participant selection

The participants were invited by email and phone. We wanted to include a wide range of physicians with varying backgrounds in our sample to ensure that we captured a wide range of perspectives on the topic under investigation. The sample consisted of physicians from a variety of specialities (e.g., internal medicine, surgery and psychiatry). Furthermore, a wide age group (from 27 to 65 years old) was represented to ensure varying seniority. The sample consisted of 9 males and 12 females and half of the sample were undergoing specialist training, while the other half were senior consultants (Additional file 1: Table S1). Data saturation [19] was reached halfway into the interview process. This implies that, after approximately 10 interviews, no new themes, findings, concepts or problems emerged. The remaining interviews ensured breadth and depth in the range of opinions and representation on the issue under investigation, for instance in relation to gender, speciality and seniority. The remaining interviews also contributed to supporting our initial findings.

### Data collection

The semi-structured interviews carried out during a three-month period in 2013. The interviews lasted from 45 to 90 min and were audiotaped. The participants were asked to provide a brief summary of their education, their career as a physician and their current position. Please refer to the Interview guide in Additional file 2.

As we were interested in exploring in depth the structural, cultural and individual aspects of sickness presence, the respondents were first asked to describe how they perceived the issue of sickness presence (e.g., “What does it mean to be ill as a physician?”, “Do you have any personal experiences with going to work when ill?” and “How did you find going to work when you were ill?”). Secondly, we asked about the general perceptions of sickness presence in their organisation (e.g., “What are the ideals/principles (formal as well as informal) surrounding the issue of sickness presence in your ward?”, “What are the consequences of sickness presence?”). Here, we were particularly interested in revealing positive as well as negative aspects of sickness presence (e.g., “Can sickness presence potentially have a positive impact on illness?” and “Are there any barriers in the organization that contribute to sickness presence when you would rather have stayed at home?”). Finally, the interviews included issues of potential means to alleviate the negative consequences of sickness presence (“Do you want something to be done about the issue of sickness presence in your organisation?”). Two of the authors, one of whom is a physician, carried out the interviews. The interview guide was not pilot-tested but, following responses we received in the process of interviewing, some questions were reframed, and some questions were more focused upon than others. We also followed up on experiences that appeared to be significant for some respondents to see if other respondents shared the same experience.

### Data analysis

The interviews were transcribed verbatim by two student assistants. Identifiable information, including name of the participants or other persons referred to in the interviews, department and age, was removed from the transcript and each interview was assigned a random number before analysis. Each author coded and analyzed the data material separately. The authors then compared and discussed themes and their content, in order to reconcile discrepancies and reach consensus to ensure the validity of the interpretations. The software program NVivo [20] was used in the analysis. Data analysis followed the three steps of systematic text condensation [21]. Firstly, as the aim of our study was to build on previous studies on sickness presence, the analysis was largely top down, and theory-driven. This implies that we adopted our theoretical and empirical knowledge about the concept of sickness presence as a starting point. Furthermore, we wanted to explore organizational, cultural and individual aspects surrounding sickness presence, and to open up positive as well as negative perspectives associated with each of these three aspects. On a practical level, we started out by reading through the interview transcripts on several occasions and isolated passages that involved content about the participants’ thoughts and experiences regarding presenteeism. We then coded this text openly. During this process it became evident that organisational, cultural and individual factors were closely intertwined, and with the notion of illness, and the process of determining whether one was ill or not. We therefore decided to distinguish ‘evaluation of illness’ as a separate issue. Hence, we ended up with four general themes that were adopted as templates in the next level of analysis, where we structured the text through isolating passages that revolved around cultural and individual issues, and evaluation of illness, respectively. Then, we reread the isolated passages (themes) and formed a set of codes that represented the different ways in which the issue of sickness presence was understood and described, including causes and consequences within each of the four themes. For instance, in the passages that revolved around the organisational factors theme, it was evident that senior physicians experienced different triggers for sickness presence than younger, more inexperienced physicians. This resulted in the codes “senior physicians present due to irreplaceability” and “inexperienced physicians present to attend potential learning situations”. Thirdly, we looked for patterns, similarities and differences in the codes that had been formed within each of the four themes.

## Results

The aim of the study was to explore the ways in which the issue of sickness presence was perceived and experienced by hospital physicians, including positive as well as negative perspectives. Following the data analysis we came up with four themes, all of which were related to some extent: 1) Evaluation of illness, 2) Organizational factors, 3) Professional culture, and 4) Individual factors. We were not able to identify any particular differences in these themes in terms of specialty or gender. Furthermore, it was evident that overall the physicians in our sample tended to reflect on issues related to all four themes, and referred to the simultaneous presence of positive as well as negative dimensions of sickness presence. The themes will be elaborated on below followed by sample quotes. Please refer to Additional file 3: Table S2 for correspondence between sample quote and the results.

### Evaluation of illness

In general, it appeared that physicians had a high threshold for sickness absence (Q1, Q2). Physicians also reported that on some occasions presenteeism was associated with positive consequences and was a remedy for poor health and beneficial for their own recovery (Q1, Q5). In contrast, it was also evident that physicians on several occasions found it difficult to evaluate their own medical conditions, which implied that they ended up attending work with illnesses that they had initially evaluated as having an adverse impact on themselves, and potentially their patients (Q1, Q2, Q3, Q4). Discretion was exercised to decide whether a medical condition was considered legitimate, and the term “respectable illness” was something that came up in interviews. Legitimate or respectable illness included critical physical conditions such as stroke, cardiac infarction and serious back pain that led to severe reduction in capability in their daily functioning. In addition, infections and contagious diseases in general and gastrointestinal infection in particular, were regarded as legitimate. It was also evident that chronic illness and mental and psychological conditions were not legitimate reasons for absence from work. When considering the legitimacy of a condition the concern for patients carried more weight than concern for the physician’s health (e.g. the risk of infecting patients). This was particularly the case when patients had travelled from afar, had waited for a long time and/or if the competence of the particular physician was considered critical for the patient. In a similar vein, many considered absence due to sick children was more legitimate than absence due to their own illness. Attending work while ill was largely experienced negatively, for instance physicians reported that this potentially led to exhaustion and prolonged recovery.

### Organizational factors

Physicians indicated that organisational factors affected evaluation of their own illness, as well as the decision to attend work while ill. At the ward level, physicians reported that sufficient staffing on their ward and a supportive departmental manager that worked out flexible solutions to arrange for absence when they, or their children, were ill prevented presenteeism. In contrast, physicians reported that working in a speciality, and ward, that was characterized by high work pressure, insufficient staffing and managers that were less supportive increased presenteeism (Q7). Consequently, physicians reported that they preferred being “sent home” after having shown up at work with an illness, rather than calling in sick. For the same reason they also preferred returning to work prior to full recovery. Some of the physicians were also concerned about the wider political and financial situation surrounding the organization and management of the hospital (Q8, Q9, Q11). Here they perceived increasing work pressure and demands on the wards, along with the lack of a support system in the event of sickness presence and long-term consequences of this such as mistakes and patient injury (Q10). Regardless of a positive or negative organizational climate, presenteeism often emerged because of irreplaceability (Q6). This was particularly the case for senior physicians with specific competencies and in wards where work was organized around teamwork. In general, the threshold for reporting ill was generally high in relation to night duty, weekends, holidays and when working in outpatient clinics. For younger and more inexperienced physicians in particular, opportunities for carrying out certain procedures that were rare and represented important learning situations represented a structural trigger to attend work while ill. There was also the issue of residents employed in temporary positions, which increased presenteeism as a strategy for them to prove their irreparability. It was a striking finding in our data material that none of the respondents in our study (except for one) were familiar with, or seemed to have been informed about, the formal policy and procedures for working with contagious symptoms at the hospital.

### Professional culture

Organizational structure also affected professional culture in the sense that the structure promoted and sustained a certain loyalty among physicians whereby sickness presence was motivated by not wanting to let colleagues down (Q12, Q14). This was something that contributed to creating social bonds at work where physicians cared for one another. The professional culture was characterized by a general view that their work was important and meaningful. Consequently, presenteeism was not solely perceived as having detrimental effects on the physicians’ health and recovery, or those of their patients. Some physicians described that they preferred to work through an illness rather than stay at home, because they perceived that staying at home would not improve their health condition. Furthermore, sickness presence was generally associated with positive feedback from colleagues and managers, something that promoted a positive self-image, and increased well-being and job satisfaction for physicians who worked through an illness. Unfortunately, there were also negative contrasts to this culture: as the standards for presenteeism were high, sickness absence was generally frowned upon, and seen as a sign of weakness and a lack of reliability and loyalty to colleagues (Q14, Q15). Illness absence could ultimately lead to negative responses from colleagues as well as ward managers. For instance, there was extensive storytelling going on in the ward in which extreme cases of illness absence were shared, typically by more senior physicians, where the ill person was described in very positive and heroic terms (Q13, Q16, Q17, Q18). However, these stories did not necessarily induce respect and admiration from (younger) colleagues or patients. Consequently, though sickness presence worked as a strategy to provide a positive self-image in the group, it also had some destructive side effects.

### Individual factors

Individual factors were also relevant in relation to sickness presence in terms of personality, upbringing, sense of work ethic and perspectives on life and work. Some of the participants described how the tendency to work at any cost was related to their sense of self-worth (Q19, Q20, Q21, Q22). Others described a strong work ethic and a high threshold for calling in sick for school/work was the core of their upbringing. Some of the participants reported having parents who were physicians, which implied socialization into the “medical perspective” on health, illness and sickness presence. This had some positive connotations as several of the physicians reported a particular view on life that enabled them to see themselves, their work and their illness in “the bigger perspective”(Q23). For instance, continuously encountering patients in poor health affected their perspectives on illness in general and potentially made their own health issues (e.g. infections) seem minor. Work was regarded as an inherent part of their life and fulfilled their strong need to contribute to society (Q24). This implied that “walking the extra mile” was experienced as deeply meaningful, and that physicians were internally motivated to do so. At the same time, work was often regarded as beneficial to their own health and something that contributed to a positive self-image. Working while sick was seen as something that exhibited devotion, a sense of responsibility and competence.

Some of the physicians also reported having developed coping strategies for themselves over time that served to ease the inherent pressures of work and subsequently to avoid illness absence/presence in the future (Q25, Q26).

## Discussion

The current study shows that sickness presence among physicians was associated with the evaluation of illness, structural, professional and individual factors, all of which were associated with positive as well as negative issues. One of the main findings in our study is that there are positive as well as negative driving forces related to sickness presence, and that these forces can operate simultaneously. For instance, a physician experiencing some kind of illness that should have prompted absence from work may experience attendance pressure due to a lack of medical staff. However, this physician may at the same time also experience joy and satisfaction in relation to helping a patient with specialized medical treatment – a positive experience that is enhanced by the knowledge that cancelling will cause delays and suffering for the patient. In contrast to Johns [1], who emphasized the relative influence and weighing of positive and negative factors in his model on presenteeism, the current study shows that presenteeism among physicians is reinforced by accompanying and at times contrasting positive and negative factors. This effect can potentially lead to a situation where physicians find it difficult to push the “stop button” until stress manifests itself in severe mental and somatic symptoms that eventually force them to focus on their own health and well-being. This is in line with studies on physicians where it was evident that physicians continue to work even though they show profound sings of burnout and psychological ill health [22].

Another important finding in this study was that physicians were surprisingly insecure and indecisive in the evaluation of their own symptoms in contrast to the certainty they exhibited in the diagnosis of patients. According to Johns’ model [1], the onset phase includes the evaluation of illness. Accordingly, it assumes that individuals are capable of assessing their symptoms in terms of severity, and that context comes in to play in less extreme medical cases. However, the findings of this study may suggest that the “chain of events” is not necessarily that straightforward for physicians, as they often seem to disregard or trivialize the nature and severity of symptoms, something that often results in sickness presenteeism, even in cases where physicians are very ill. Future studies should focus on the evaluation of symptoms in the onset phase of a health event, and how this in turn can contribute to presenteeism directly and indirectly with possible severe consequences for the individual physicians and the delivery of health-care services.

The current study confirms a variety of both positive and negative contextual and personal factors that have been shown to promote presenteeism in previous studies. The most prominent positive factors were increased experience [23, 24], work joy, self-image, self-confidence and professional identity [15], support and positive leadership. The negative factors were permanency of employment [25], poor organizational structure and policy [1] and replaceability in terms of lean staffing, high specialization or perceived unfairness to colleagues [12, 16]. Meanwhile, some factors such as high job demands and professional culture [14, 16, 26] contributed positively and negatively to presenteeism depending on the situation. In addition, the relevance of these factors also varied according to seniority. The latter highlights the highly situation-specific and complex nature of presenteeism.

### Strengths and limitations

The participants were recruited through snowball sampling via the authors’ network and participants’ recommendations. Consequently, it can be argued that our sample was skewed in favour of participants with a profound interest in the topic under investigation. However, there was nothing in our data that suggested that this was the case. In general, participants appeared reflective and willing to reveal personal stories that provided a balanced view, including positive and negative aspects. Furthermore, the fact that saturation was reached relatively soon during data collection confirms that presenteeism is a well-known phenomenon among hospital physicians, and that relevant categories and the variability and relationship between them are validated across gender and seniority among the participants.

One of the interviewers in our study was a physician, something that can be considered strength in guiding interview questions onto relevant topics, and in recognizing the participants’ work situation and points of reference. However, this position could also result in a biased relationship with the informants and their work situation, and thereby prevent a sufficiently reflective view. To prevent such bias the other authors had a non-clinical background, but were knowledgeable about the organization and professional group under investigation and held extensive expertise on the topic, as well as the qualitative methodology. Hence, it can be argued that the mix within the research group contributed to a balanced and critical interpretation of findings.

## Conclusions

The study shows the complexity of sickness presence among physicians and how presenteeism may occur from a lack of evaluating health symptoms, and increased by accompanying, and at times contrasting, positive and negative factors. The complexity of contributing factors can explain the persistent high prevalence of this phenomenon among physicians and the difficulties in changing this behaviour. However, the current study points out the factors that should be cultivated in order to facilitate positive work attendance and the organizational, professional and individual factors that should be amended to reduce the adverse consequences of presenteeism. The organization should facilitate a positive work environment, sufficient staffing and predictability in employment to prevent negative attendance pressure. It is also important to communicate formal policy on illness behaviours to avoid the adoption of discretion when the physician is ill. Because professional culture appears to be prominent for presenteeism, leaders and senior physicians as culture bearers can be regarded as particularly responsible when it comes to renewing a professional culture that diminishes the deep-rooted influence of “self-sacrificing heroes” among senior physicians, in favour of a culture where the link between healthy physicians and productive organizations is recognized. Medical schools need to go beyond the medical curriculum and promote and teach graduates the skills necessary to recognize distress and to develop strategies to promote their own well-being. This will promote professionalism, and lay the foundation for resilience and work satisfaction over the course of their career.

## Additional files

Additional file 1: Table S1. Participant characteristics. (DOCX 21 kb)
Additional file 2: Attachment 1. Interview guide, translated from Norwegian to English. (DOCX 18 kb)
Additional file 3: Table S2. Themes and sample quotes. (DOCX 28 kb)
