# Supplementary material for: Why hospital physicians attend work while ill? The spiralling effect of positive and negative factors
Source: BMC Health Serv Res. 2016 Oct 5;16:548. doi: 10.1186/s12913-016-1802-y (PMC5050593; doi:10.1186/s12913-016-1802-y)
Supplement: Additional file 1: Table S1. — Participant characteristics. (DOCX 21 kb) [file 12913_2016_1802_MOESM1_ESM.docx]

**Table 1. Participant characteristics**

| **No** | **Gender** | **Age** | **Title** | **Type of employment** |
| --- | --- | --- | --- | --- |
| 1 | Male | 40-49 | Senior | Temporary |
| 2 | Male | 40-49 | Senior | Permanent |
| 3 | Male | 20-29 | Resident | Temporary |
| 4 | Female | 30-39 | Resident | Temporary |
| 5 | Female | 40-49 | PhD student | Temporary |
| 6 | Male | 30-39 | Resident | Permanent |
| 7 | Female | 30-39 | Resident | Temporary |
| 8 | Male | 40-49 | Senior | Permanent |
| 9 | Male | 30-39 | Senior | Temporary |
| 10 | Female | 30-39 | Resident | Temporary |
| 11 | Female | 30-39 | Resident | Unknown |
| 12 | Female | 50-59 | Senior | Permanent |
| 13 | Female | 30-39 | Resident | Temporary |
| 14 | Female | 30-39 | Senior | Temporary |
| 15 | Male | 30-39 | Resident | Permanent |
| 16 | Female | 40-49 | PhD student | Temporary |
| 17 | Male | 60-69 | Senior | Permanent |
| 18 | Female | 40-49 | Senior | Permanent |
| 19 | Female | 30-39 | Resident 50%  Researcher 50% | Permanent |
| 20 | Male | 30-39 | Resident 50%  Researcher 50% | Temporary |
| 21 | Female | 40-49 | Senior | Permanent |
