# Supplementary material for: Why hospital physicians attend work while ill? The spiralling effect of positive and negative factors
Source: BMC Health Serv Res. 2016 Oct 5;16:548. doi: 10.1186/s12913-016-1802-y (PMC5050593; doi:10.1186/s12913-016-1802-y)
Supplement: Additional file 2: — Attachment 1. Interview guide, translated from Norwegian to English. (DOCX 18 kb) [file 12913_2016_1802_MOESM2_ESM.docx]

**Interview guide**

**Background questions**

We will start by asking a few questions about yourself:

- What is your educational background? Where did you study? When?
- Could you please tell us a bit about your professional experience? Previous conditions of employment?
- What is your current position? Permanent vs. temporary employment? If temporary, for how long? Part time or full time?
- Are you employed at the university or elsewhere in addition to your current position?
- Marital status? Profession of potential partner? Part time or full time?
- Children? How many? Age of potential children?
- Could you please describe your everyday job? Research, teaching, attending patients, administrative tasks.
- If involved in research/teaching, could you please describe your projects and your role in this work? Specialist training?
- How many hours do you work per week? How much of this time is extending your work contract?

**Personal views on, and experiences with, sickness presence**

- Could you please tell us what your thoughts are regarding going to work while ill? Feel free to apply your own as well as your colleagues’ experiences.
- How would you describe a physician that is ill? What is your attitude to sickness absence?
- What kind of illness do you consider that it is ok/not ok to attend work with?
- Did you ever experience attending work while you were sick/your child was sick?
- What kind of illness? How often did this happen?
- Could you please tell us a bit about how you experienced attending work while sick? Please describe specific incidents and situations.
- What would you say prevents you from absence while sick?
- What are your thoughts on being absent from work due to children being sick? How do you solve this situation on a practical level?
- Do you feel responsible for solving practical problems at you ward in relation to your absence when ill? Or do you feel confident that this is something that you don't have to worry about when ill because it is sorted out at the ward level? Please explain.

**Thoughts and views on the organization**

- What do you consider to be the ideals/principles surrounding sickness presence at your ward?
- How would you say that colleagues being absent from work due to illness are perceived at your ward?
- Are there any formal guidelines for sickness absence at your hospital? In case there are, what are they?
- Informal guidelines for sickness absence at your ward?
- Who do you talk to when considering whether to stay at home or not? Who would you say influences your decision of whether to stay at home or not the most?

**Causes of sickness presence**

- Why would you say you decide to attend work in situations where other employees would potentially stay at home?
- How do you experience going to work while sick?
- How would you say that sickness presence affects your work? Your relationship to your colleagues? Motivation? Family life?

Negative aspects surrounding sickness presence

• Do you attend work while sick due to your employment situation? (Temporary position, type of position etc.).

• Other organizational aspects? (e.g. shift arrangements, formal/informal guidelines for absence etc.)

• Professional culture/identity as a physician?

• Personal aspects

• Family situation

• Financial aspects

Positive aspects surrounding sickness presence (the joy associated with working)

• Did you ever experience that work has represented something positive while ill? Please explain and describe specific situations and incidents.

**Consequences of sickness presence**

- Please describe the consequences associated with being absent from work? (From your experience, relationship with colleagues, patient relations etc.)
- If you ever attended work while ill, how would you say that you were affected by this? How was your job situation affected? (Psychological, physical, relationship with family, colleagues, patients)
- What are the consequences if one of your colleagues attends work while sick? (Psychological, physical, relationship with family, colleagues, patients)

**Change initiatives**

- Would you say that there is a wish to do something about the issue sickness presence within the medical community?
- If so, do you have any thoughts on potential measures to reduce sickness presence? From an individual perspective, managerial perspective, organizational perspective, from the perspective of professional representatives.
- What would make it difficult to do carry through potential measures? What would help to carry through potential measures?

**Closure/Summary**

- Is there anything you would like to add before ending this interview.
