# Supplementary material for: Why hospital physicians attend work while ill? The spiralling effect of positive and negative factors
Source: BMC Health Serv Res. 2016 Oct 5;16:548. doi: 10.1186/s12913-016-1802-y (PMC5050593; doi:10.1186/s12913-016-1802-y)
Supplement: Additional file 3: Table S2. — Themes and sample quotes. (DOCX 28 kb) [file 12913_2016_1802_MOESM3_ESM.docx]

**Table 2**

Themes and sample quotes.

| **Evaluation of illness** | **Organizational structure** | **Cultural factors** | **Individual factors** |
| --- | --- | --- | --- |
| Q1: Physician 14: *“I really was not feeling well, but I thought “I have to go to work”, so I went, and felt really ill, I had to rest in between patients, but at some point the medical secretary approached me and said “lets do some tests”, and you know my CRP level was above 150, approaching 200 […] it’s this feeling, “am I really ill? Maybe not? Well, I just have to go to work and wait and see.” […] Where it the boundary? You’re used to working really hard, although you’re exhausted, it’s difficult to know when to stop. It’s a lot easier to define on behalf of other people.[…] sometimes I have to ask my colleagues, could you just listen to me, and tell me what you think? Do I need to start taking antibiotics? I need help, that someone else hears me out […] but at the same time (going to work while ill), you feel that you’re needed, you meet other people […] you feel valuable.”*  Q2: Physician 18: *“A long time went by until I realised I was ill, and even longer until I went to see my doctor and accepted ill leave […] before I got ill I thought that if I ever experience a cardiac infarction, stroke, break a leg, I’ll not go to work, but after I got ill, and saw the condition in which I went to work with, then I realised that I might actually end up going to work even with a cerebral infarction.. I don’t know.”*  Q3: Physician 19: “*Well, if I thought I’d represent a danger to the patient I wouldn’t have done it (worked while ill), then I’d for instance ask one of my colleagues to do the surgery, and I would assist. However, I’m not sure I would have wanted to receive surgery from a physician in that condition [..] I would have been worried that this person did not have the abilities to do the right judgement.”*  Q4: Physician 11: *“You know it’s about your ability to respond, and react, and observing myself in this condition (being ill) made med frightened, it was scary, icky.. So my husband came with me, he drove the car, so that I was able to carry out my shifts.”*  Q5: Physician 1: *“It was frustrating (suffering from chronic illness), but at the same time I did not experience attending work as entirely negative […] It’s about feeling useful, that’s a driving force. […] you know staying at home ruminating, being at work is better, then you can focus on the suffering of other people, disregarding your own (laughing) […] It feels good to leave the role of a ill person behind, feeling that you have something to give back to society […] I feel that I don’t get any worse by going to work, and not any better by staying at home.”* | Q6: Physician 9: *“When you’re on a temporary contract, well then you start thinking that you want to avoid everything that is negative. Overtime work, illness absence, are you away a lot? Do you need a lot of help? You want to solve problems, on your own, right, show that you’re good at your work.”*  Q7: Physician 7: *“I’ve gone to work when I’ve been so ill that I couldn’t even tie my own shoe laces […] You have no choice, there are nobody there, everyone are under so much pressure, not enough staff, the tempo is so high, on all functions.”*  Q8: Physician 8: *“Well, the waiting lists have to go down, you have to keep deadlines […] you have to follow recommendations from the government, or there will be financial sanctions, so there’s a vicious circle, we have to change routines, increase the pressure, work more efficiently, everything is supposed to go fast, you’re supposed to be present, and if you’re not there will be consequences [..] and this makes the organization less creative, I think that’s the biggest problem, you get less flexible and creative, and it becomes difficult to do something about the structure, you sort of accept the situation the way it is, and do your best to survive.”*  Q9: Physician 1: *“I’ve experienced that people that have gone over the edge, you know they’ve totally collapsed, and we should have had a system where they’ve been taken care of, before things got out of hand […] well, we’re a large organization, and even if you’re a physician, and you perceive that you have a lot of responsibility, on a personal level, you’re still just a small wheel in a big machinery […] it’s a premise that you tolerate high pressure. You’re supposed to go the extra mile, tolerate high work pressure over time, too much pressure, and then typically, you do a mistake, with minimal support. So if something goes wrong, you’re pretty much on your own.”*  Q10: Physician 11: “*The more vulnerable you are the less likely you make the right judgement of yourself.”*  Q11: Physician 18: *“In our job, it’s not enough just to show up, there are some requirements, we have to be creative, we have to invest as a human beings, provide something additional, and our employer benefits from our engagement ,benefits from the things that make us go to work a lot of the time. But we’re not being validated for this, we’re not appreciated, and constantly more is asked from us.”* | Q12: Physician 1: *«The most important driving force is the loyalty you feel towards your colleagues [..] I think we should be honest about that.. And the patients…»*  Q13: Physician 9: *«If someone is away a lot, from shifts, it doesn’t take long until it’s talked about […] well, we do have a responsibility, physicians create their own work environment […] You have these stories, like «what? On ill leave while you’re pregnant? You know what, when I was pregnant I worked until the last contractions». Like «my water broke when I was on my shift.» And that’s the kind of stories that are being told in very positive ways, and that sets a kind of standard, in our working environment.»*  Q14: Physician 11: *«It’s not the managing director of the hospital I feel sorry for, right, it’s my colleagues […] Being ill… it’s not something positive…[..] I’m very concerned that people know that my threshold for staying at home is very high. So when I have been absent I try to make sure it’s still showing that I’m ill when I get back to work […]»*  Q15: Physician 12: *“I don’t want to be perceived as someone who breaks easily […] there is this contempt for weakness. […] it’s a cultural thing, plus we’re generally under a lot of pressure.”*  Q16: Physician 6: “*You look up to them, sort of, you don’t want to be weaker than them, when you see that they, for instance you observe a chief physician come in to work with crutches and a plastered leg, of course, this is contagious, then it’s not ok to stay at home in the same condition.”*  Q17: Physician 13: *“It’s reinforced in our environment, not explicitly, but working until you’re very tired is high status, we talk about this in an admiring way, it’s a cultural thing, well, a bad culture […]* *It’s devotion gone wrong.”*  Q18: Physician 1: *«He appeared to be on strong medications (colleague), even our patients thought it was striking.”* | Q19: Physician 21: *«It’s about standing tall, feeling worthy. It’s rooted deep. I’ve sometimes experienced that it can be destructive to have these kinds of expectations to oneself . Dragging yourself to work when you should have stayed home. It would have been a lot healthier if I could have told myself that «you know what, I’m feeling really ill, I just have to stay home» and still feeling like a good person. […] we’re so vulnerable, because our self-image is so closely connected to our accomplishments […] it’s about how we’re thinking about this, inside our heads.»*  Q20: Physician 13: *“As a group we’re pretty fulsome, we want to please.”*  Q21: Physician 1: *«You’re not supposed to have problems.»*  Q22: Physician 9: *«It’s very uncomfortable calling in ill. Why? I think it’s an admission of weakness.»*  Q23: Physician 15: *“ I go to work because I enjoy being at work […] Well, you’ve had some epiphanies throughout life, right, life is filled with uncertainties , the only thing you know is here and now, you never know what tomorrow brings, so making the best out of the situation, right, the whole project is a palliation […] we should be happy that work is part of our life.”*  *Q24: Physician 18: “I feel committed to society, I feel obliged to contribute. I have this qualification, and I think we should all do our share. I’ve not come to this earth to do nothing, I want to utilize the abilities I’ve been handed to do something…I have a sense of duty, but I feel joy, I feel privileged […] a sense of duty is not negative to me.”*  Q25: Physician 14: *“It’s important to learn to care of yourself, you know, “taking care of number one” […] for instance, when you wash your hands, controlling all these negative thoughts, try to be present, in the moment.”*  Q26: Physician 16: *“I can choose not to worry about certain things.”* |
